# Pragmatic trials of non-NHS interventions: experiences from a Randomised Controlled Trial of the Strengthening Families 10-14 UK Programme (SFP10-14 UK)

**DOI:** 10.1186/1745-6215-12-S1-A98

**Published:** 2011-12-13

**Authors:** Jo Holliday, J Segrott, H Rothwell, C Phillips, K Hood, Z Roberts, J Scourfield, S Murphy, D Foxcroft, P Daniels, L Moore

**Affiliations:** 1 Cardiff Institute of Society and Health, School of Social Sciences, Cardiff University, 1-3 Museum Place, Cardiff, CF10 3BD, UK

## Background

Pragmatic trials of public health interventions outside the NHS are relatively scarce, much needed and face particular challenges. These include funding, of intervention costs in particular; trial implementation in professional and organisational cultures unused to randomised trial procedures, including randomisation, maintaining the counterfactual, recruitment; and relevance of findings for and translation into policy and practice.

## Objectives

The current NPRI funded trial of SFP 10-14 UK is presented as a case study to discuss these issues, solutions and remaining barriers. The SFP 10-14 UK programme aims to strengthen areas of family life that protect against substance misuse, for example, parenting, communication, and young people’s resilience skills. The SFP 10-14 UK is being delivered by statutory and voluntary agencies in six local authority areas across Wales, and is offered to mixed groups comprised of families from the general population, and families who may experience/present challenges within a group setting.

## Methods

The trial aims to recruit 748 families, 374 of whom will be randomised to receive the usual services available to families within their local area. 374 families will receive the SFP 10-14 UK in addition to usual care. Families are identified by staff employed within the statutory services and voluntary sectors and referred to embedded research staff for recruitment.

## Results

Challenges encountered related to a lack of awareness of the randomised trial as a research paradigm among staff and key referring agencies, related concerns about the ethics of randomisation and the maintenance of the counterfactual among the usual care group, and challenges regarding the maintenance of recruitment and intervention fidelity. Whilst a challenge in itself, partnership working with delivery agencies, programme trainers, and the Welsh Assembly Government at all stages of the development, funding and conduct of the trial has proved an important strategy to overcome these issues.

## Conclusions

This trial seeks to generate evidence on the effectiveness and cost effectiveness of the SFP10-14 UK which is of direct relevance to policy makers, commissioners and practitioners. The trial highlights that strategic partnership working, the winning of ‘hearts and minds’ regarding the ethics and operationalisation of randomisation, and maintaining the balance between internal and external validity are key areas of focus for the successful conduct of pragmatic trials in non-NHS settings. The lessons learnt from its implementation will be important for future multi-sector/agency policy trials and for role out of the intervention if found to be efficacious.

